# Doxycycline Impairs Mitochondrial Function and Protects Human Glioma Cells from Hypoxia-Induced Cell Death: Implications of Using Tet-Inducible Systems

**DOI:** 10.3390/ijms19051504

**Published:** 2018-05-17

**Authors:** Anna-Luisa Luger, Benedikt Sauer, Nadja I. Lorenz, Anna L. Engel, Yannick Braun, Martin Voss, Patrick N. Harter, Joachim P. Steinbach, Michael W. Ronellenfitsch

**Affiliations:** 1 Dr. Senckenberg Institute of Neurooncology, University Hospital Frankfurt, Goethe University, Schleusenweg 2-16, 60528 Frankfurt am Main, Germany; Anna-Luisa.Luger@kgu.de (A.-L.L.); ben@sauer-web.de (B.S.); nadja.lorenz@googlemail.com (N.I.L.); anna.engel@outlook.com (A.L.E.); martin.voss@kgu.de (M.V.); joachim.steinbach@med.uni-frankfurt.de (J.P.S.); 2 German Cancer Research Center (DKFZ) Heidelberg, Germany and German Cancer Consortium (DKTK), Partner Site Frankfurt/Mainz, Theodor-Stern-Kai 7, 60590 Frankfurt am Main, Germany; 3 Institute of Neurology (Edinger-Institute), University Hospital Frankfurt, Goethe University, Heinrich-Hoffmann Str. 7, 60528 Frankfurt am Main, Germany; ynck10@gmail.com (Y.B.); patrick.harter@kgu.de (P.N.H.)

**Keywords:** doxycycline, tetracycline, inducible gene expression, Tet-inducible system, hypoxia-induced cell death, mitochondria, tumor metabolism, glioblastoma

## Abstract

Inducible gene expression is an important tool in molecular biology research to study protein function. Most frequently, the antibiotic doxycycline is used for regulation of so-called tetracycline (Tet)-inducible systems. In contrast to stable gene overexpression, these systems allow investigation of acute and reversible effects of cellular protein induction. Recent reports have already called for caution when using Tet-inducible systems as the employed antibiotics can disturb mitochondrial function and alter cellular metabolism by interfering with mitochondrial translation. Reprogramming of energy metabolism has lately been recognized as an important emerging hallmark of cancer and is a central focus of cancer research. Therefore, the scope of this study was to systematically analyze dose-dependent metabolic effects of doxycycline on a panel of glioma cell lines with concomitant monitoring of gene expression from Tet-inducible systems. We report that doxycycline doses commonly used with inducible expression systems (0.01–1 µg/mL) substantially alter cellular metabolism: Mitochondrial protein synthesis was inhibited accompanied by reduced oxygen and increased glucose consumption. Furthermore, doxycycline protected human glioma cells from hypoxia-induced cell death. An impairment of cell growth was only detectable with higher doxycycline doses (10 µg/mL). Our findings describe settings where doxycycline exerts effects on eukaryotic cellular metabolism, limiting the employment of Tet-inducible systems.

## 1. Introduction

Genetic engineering has become an indispensable part of modern molecular biology research. Tetracycline (Tet)-regulated systems are commonly used for inducible and reversible gene expression in mammalian cells where tetracycline or its derivative doxycycline are the regulating agents governing transcription from genetically engineered constructs. Two principle systems with opposite responsiveness are available: Tet-on systems rely on a transactivator protein that in the absence of tetracycline/doxycycline is unable to bind to its corresponding promoter and activate transcription [1]. Tet-off systems rely on a slightly modified transactivator protein that in the presence of tetracycline/doxycycline loses its affinity to bind and activate transcription [2]. Employment of these systems for experiments requires constitutive expression of the respective transactivator protein and the presence of the gene of interest under control of a promoter with Tet-responsive elements. More recently, single plasmid systems incorporating all required DNA sequences have been developed [3]. While this is a very elegant method at first glance allowing rapid and reversible expression of a protein in an identical genetic background, the use of Tet for regulation might lead to relevant alterations in cellular metabolism.

Both tetracycline and doxycycline are commonly used antibiotics that target the prokaryotic ribosomal complex by binding to the 30S bacterial ribosomal subunit, thereby preventing the association with aminoacyl-transfer RNAs (tRNAs) [4,5,6] and exerting a bacteriostatic effect. Recent reports have already called for caution when using tetracycline and derivatives to study gene expression in mammalian cells [7] because of a potentially evolutionary conserved link between mitochondria and bacteria [8]. The mitochondrial protein translation machinery is responsible for production of 13 proteins encoded in the mitochondrial DNA, all of which are components of the respiratory complexes [9,10]. Translation of mitochondrially-encoded proteins occurs within the organelle at the mitoribosome [11] which, similar to eukaryotic or prokaryotic ribosomes, consists of a small and a large subunit (28S and 39S, respectively) [12]. It has also been reported that, at higher doses, tetracycline and doxycycline can interfere with mammalian protein synthesis [13]. Dose-dependent inhibitory effects on mitochondria are detectable across different species and tissues, suggesting a highly conserved phenomenon [7]. These effects include disturbance of mitochondrial proteostasis and function as well as disruption of global transcriptional profiles [7]. Some data already exist regarding metabolic effects of doxycycline on lactate secretion and oxygen consumption in human cancer cells including one glioma cell line [14]. However, the observed effects varied significantly among the tested cell lines and no extensive analysis of metabolic effects of doxycycline on glioma cell lines regularly used for glioma research as well as of implications on hypoxia-induced cell death has been performed so far. As “reprogramming of energy metabolism”, besides “evading immune destruction”, has become one of the two emerging hallmarks of cancer in the last decade [15], studying tumor metabolism is an important pillar in modern cancer and glioma research. Therefore, the scope of this study was to systematically analyze dose-dependent metabolic effects of doxycycline on a panel of glioma cells to gain detailed insight into metabolic effects following doxycycline treatment, especially for most of the concentrations relevant for current Tet-systems.

## 2. Results

### 2.1. Doxycycline Impairs Mitochondrial Function in Glioma Cells

**Figure 1:**
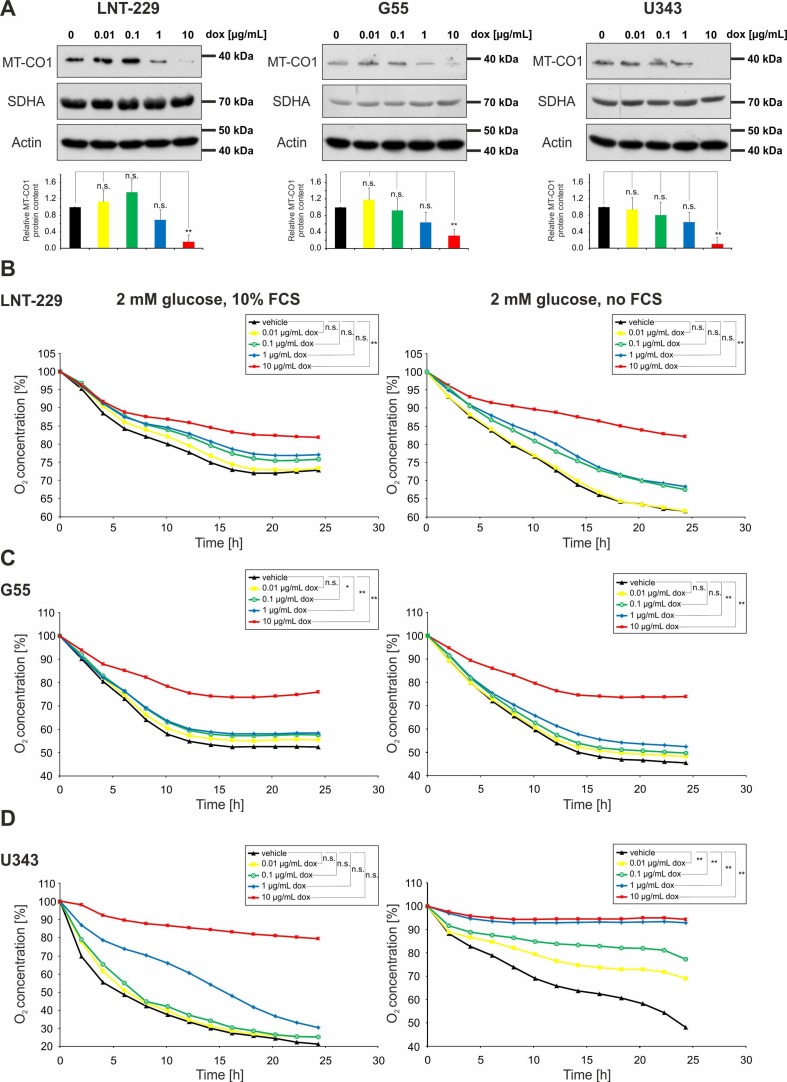
Doxycycline impairs mitochondrial function in glioma cells. (**A**) LNT-229, G55, and U343 glioma cells were incubated in serum containing (10% fetal calf serum, FCS) medium without glucose restriction (25 mM glucose) with vehicle, 0.01, 0.1, 1, or 10 µg/mL doxycycline under normoxic conditions for 24 h. Cellular lysates were analyzed by immunoblot with antibodies for mitochondrially-encoded cytochrome c oxidase I (MT-CO1), succinate dehydrogenase complex, subunit A (SDHA), and actin. Quantification of the MT-CO1 expression is shown in the lower panels (*n* = 3, mean ± S.D.; n.s. = not significant, ** *p* < 0.01). (**B**–**D**) LNT-229 (**B**), G55 (**C**), and U343 (**D**) cells were incubated in either glucose-restricted (2 mM glucose) DMEM containing 10% FCS (left panel) or glucose-restricted (2 mM glucose) serum-free DMEM (right panel) in each condition with vehicle, 0.01, 0.1, 1, or 10 µg/mL doxycycline and overlaid with sterile paraffin oil. Oxygen consumption was measured by a fluorescence-based assay and is depicted relative to the start of the experiment (*n* = 3, mean; n.s. = not significant, * *p* < 0.05, ** *p* < 0.01; for easier distinction of the curves, the standard deviation of values has been omitted in the diagrams).

Three well-established glioma cell lines (LNT-229, G55, and U343) were treated with increasing concentrations of doxycycline. Mitonuclear protein imbalance was determined as already described [7,16] by comparing the ratio of the mitochondrially-encoded cytochrome c oxidase I (MT-CO1) and the nuclear-encoded succinate dehydrogenase complex, subunit A (SDHA). MT-CO1 protein content was decreased with doxycycline concentrations of 1 µg/mL and higher in all three glioma cell lines (Figure 1A). In contrast, none of the investigated doxycycline concentrations had any effect on SDHA protein content. (Figure 1A). A concentration of 10 µg/mL doxycycline rendered MT-CO1 almost undetectable in all tested cell lines. To account for more realistic in vivo conditions for metabolic measurements, 2 mM glucose medium was used with or without fetal calf serum (FCS) for the following experiments. Under these culture conditions, MT-CO1 expression was similarly decreased in a dose-dependent manner upon doxycycline treatment in all tested cell lines (Figure S1). To test for a functional relevance of the observed mitonuclear protein imbalance of respiratory chain-associated proteins, dose-dependent effects of doxycycline on oxygen consumption were measured (Figure 1B–D). A dose-dependent reduced oxygen consumption was recorded for all tested cell lines (Figure 1B–D). In LNT-229 cells under FCS-containing conditions, a significant reduction of oxygen consumption was only observed for 10 µg/mL regardless of serum (Figure 1B). In G55 cells, reduced oxygen consumption was detectable at 0.1 µg/mL under 10% FCS (Figure 1C, left panel). Under serum-free conditions an impaired oxygen consumption was detected above 0.1 µg/mL (Figure 1C, right panel). In U343 cells, statistical significance was not reached even though a clear decrease in oxygen consumption was observed under 1 and 10 µg/mL doxycycline in the presence of FCS (Figure 1D, left panel). Withdrawal of serum led to a statistically significant inhibition of doxycycline on oxygen consumption, which became detectable already at a concentration of 0.01 µg/mL doxycycline (Figure 1D, right panel).

### 2.2. Doxycycline Induces Glucose Consumption in Glioma Cells

**Figure 2:**
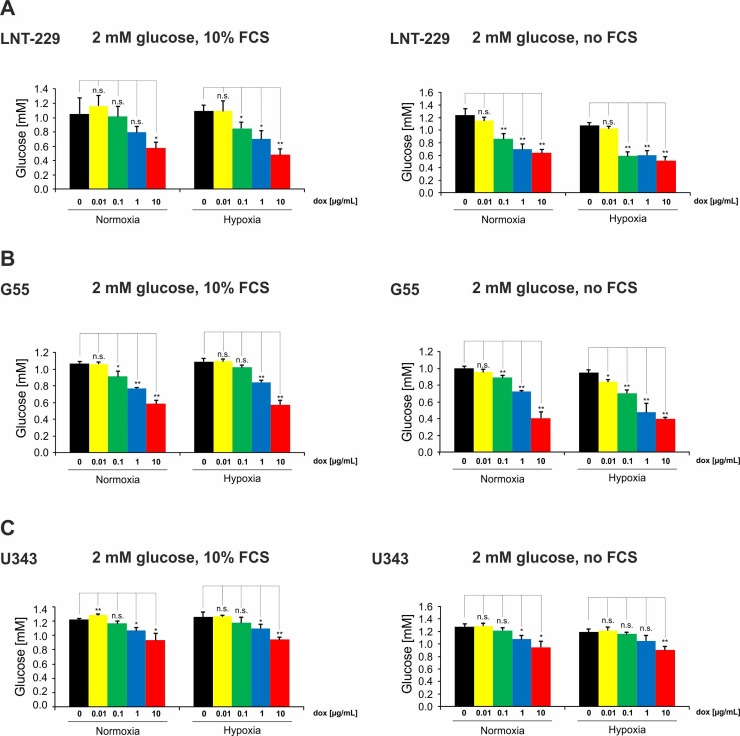
Doxycycline induces glucose consumption in glioma cells. LNT-229 (**A**), G55 (**B**), and U343 (**C**) cells were incubated either in glucose-restricted (2 mM glucose) DMEM containing 10% FCS (left panel) or glucose-restricted (2 mM glucose) serum-free DMEM (right panel) in each condition with vehicle, 0.01, 0.1, 1, or 10 µg/mL doxycycline under normoxic (21% oxygen) or hypoxic (0.1% oxygen) conditions for 8 h. Remaining glucose was determined in the supernatant (*n* = 3, mean ± S.D.; n.s. = not significant, * *p* < 0.05, ** *p* < 0.01).

Disruption of mitochondrial proteohomeostasis with concomitant impaired oxygen consumption does limit energy supply from oxidative phosphorylation. A potentially compensatory induction of glucose consumption was observed in all tested cell lines in a doxycycline dose-dependent manner (Figure 2). There was a trend for a slightly more pronounced glucose consumption under hypoxic conditions and with serum withdrawal in LNT-229 and G55 cells: In LNT-229 cells, a significant increase in glucose consumption was observed with 10 µg/mL doxycycline under incubation with FCS in normoxia (Figure 2A, left panel). Hypoxia enhanced this observed effect, which became significant already with 0.1 µg/mL doxycycline (Figure 2A, left panel). Serum withdrawal caused an increase in glucose consumption that could already be observed with 0.1 µg/mL doxycycline under normoxic and hypoxic conditions (Figure 2A, right panel). In G55 cells, an increase in glucose consumption could be observed for concentrations of 0.1 µg/mL doxycycline or higher under incubation with FCS in normoxia (Figure 2B, left panel). In hypoxic conditions this effect was significant with 1 µg/mL doxycycline (Figure 2B, left panel). Serum withdrawal did not affect glucose consumption in normoxia. Under hypoxic conditions an increase in glucose consumption became apparent already with doxycycline concentrations of 0.1 µg/mL (Figure 2B, right panel). In U343 cells, an increase in glucose consumption could be observed starting at 1 µg/mL doxycycline under incubation with FCS in normoxia and hypoxia (Figure 2C, left panel). Serum withdrawal had no additional effect under normoxia; however, under hypoxia a significant increase in glucose consumption could be observed only with 10 µg/mL doxycycline (Figure 2C, left panel).

### 2.3. Doxycycline Can Have Converse Effects on Hypoxia-Induced Cell Death in Glioma Cells

**Figure 3:**
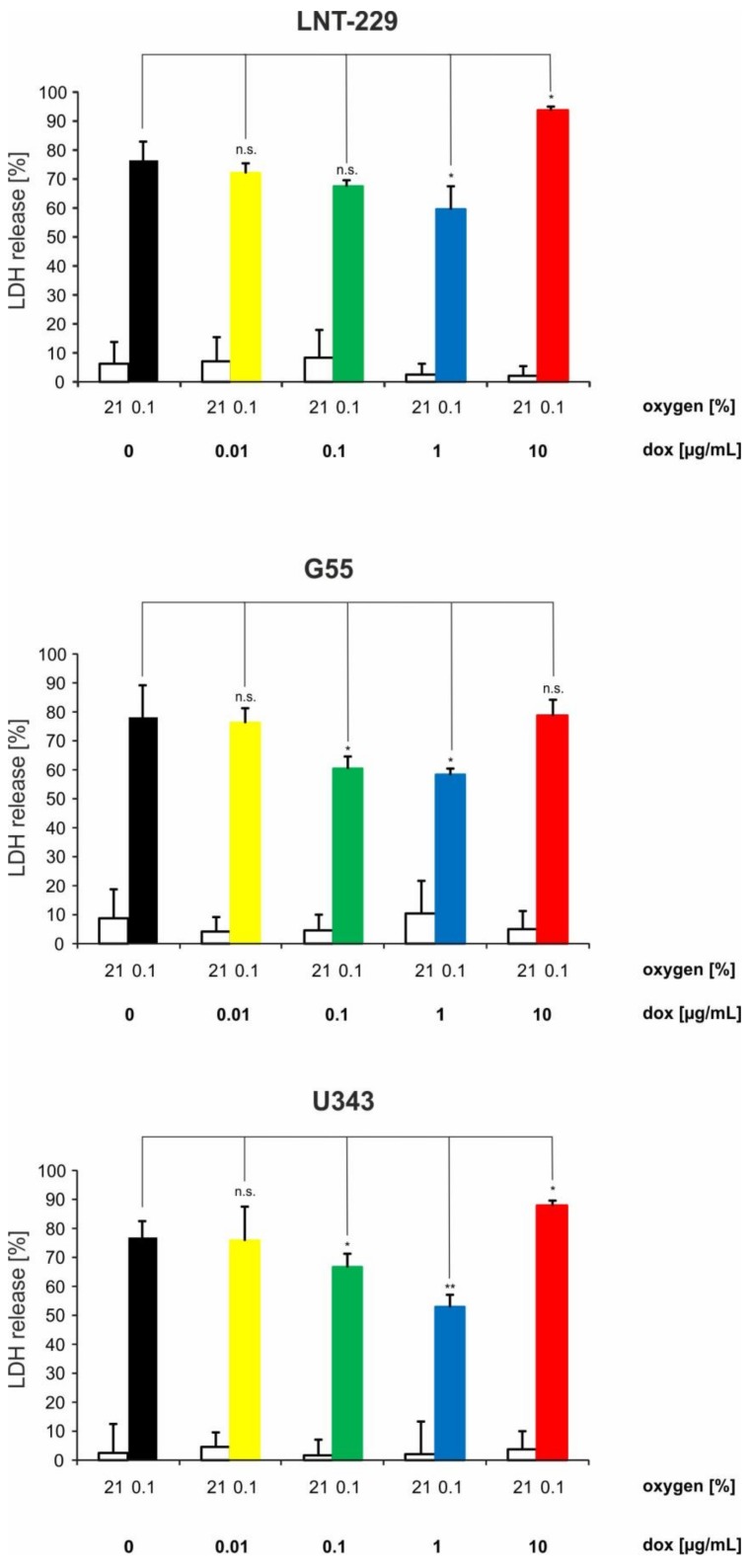
Doxycycline shows converse effects on hypoxia-induced cell death in glioma cells. LNT-229, G55, and U343 glioma cells were exposed to glucose-restricted (2 mM glucose) serum-free DMEM with vehicle, 0.01, 0.1, 1, or 10 µg/mL doxycycline under normoxic conditions (21% oxygen) or 0.1% oxygen until cell death was observed. Cell death was quantified using the LDH release assay (*n* = 4, mean ± S.D.; n.s. = not significant, * *p* < 0.05, ** *p* < 0.01).

To test whether the observed metabolic changes with reduced oxygen consumption had effects on cellular survival under glucose- and oxygen-restricted conditions, we performed cell viability measurements using LDH release as a marker of cell death. Doxycycline concentration of 0.1 as well as 1 µg/mL protected from hypoxia-induced cell death (Figure 3). In contrast, a further increased concentration of 10 µg/mL doxycycline, which is beyond commonly used doses when using Tet-systems, enhanced sensitivity to hypoxia-induced cell death, as indicated by an increase in lactate dehydrogenase (LDH) release (Figure 3). Notably, all employed doxycycline doses had no effect on cellular viability under normoxic conditions (Figure 3).

### 2.4. Doxycycline Inhibits Growth of Glioma Cells Only under High Concentrations

**Figure 4:**
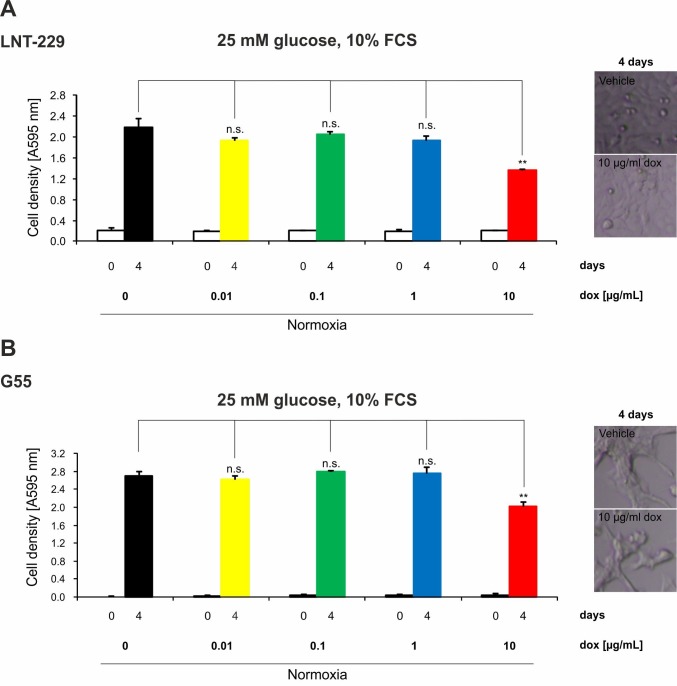

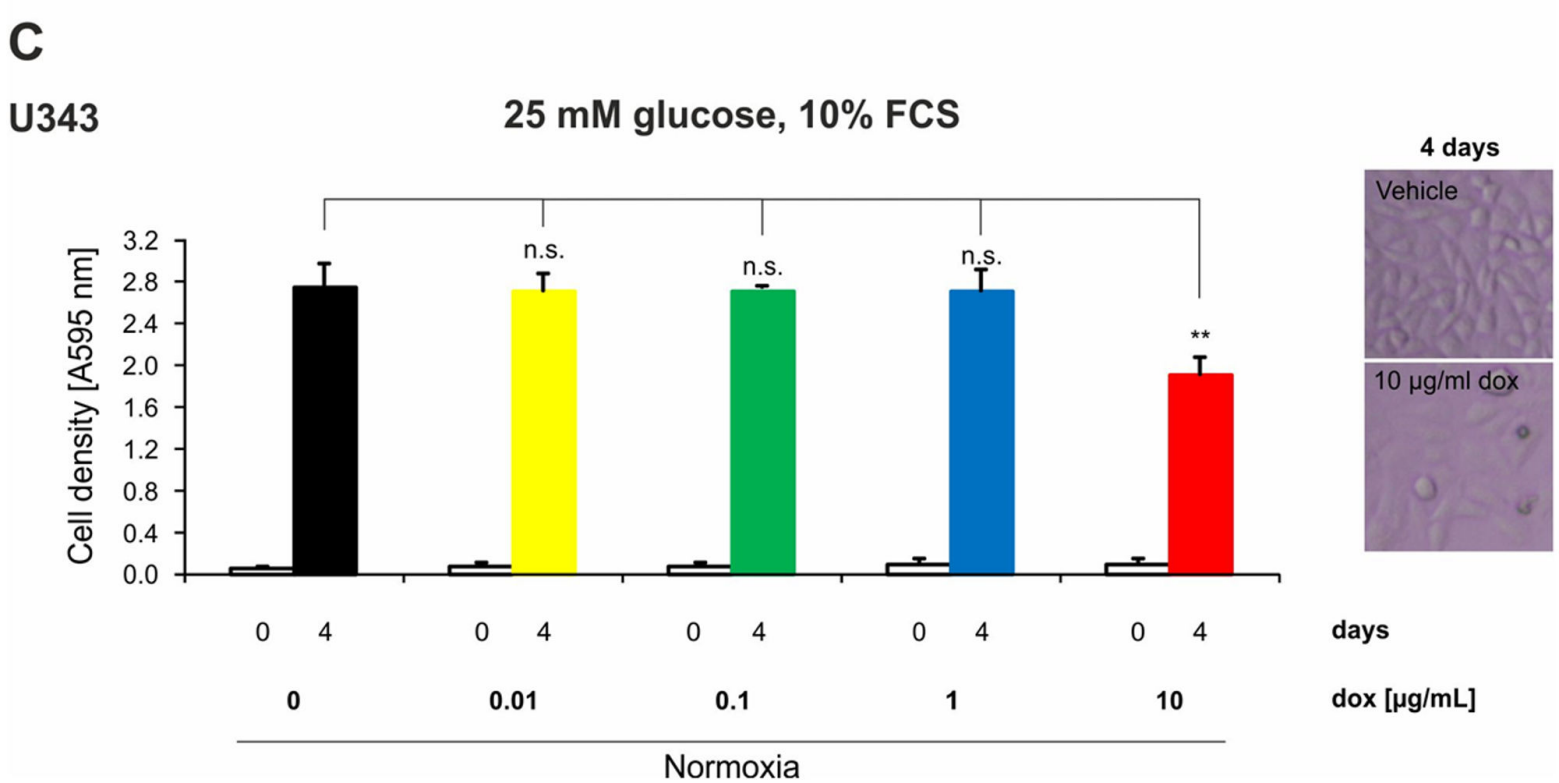
Doxycycline affects growth of glioma cells only under high concentrations. LNT-229 (**A**), G55 (**B**), and U343 (**C**) glioma cells were incubated in serum-containing (10% FCS) culture conditions without glucose restriction (25 mM glucose) with vehicle, 0.01, 0.1, 1, or 10 µg/mL doxycycline for 4 days. Cell density was measured by crystal violet (CV) staining at the beginning of cultivation and after 4 days (*n* = 4, mean ± S.D.; n.s. = not significant, ** *p* < 0.01). Representative photographs of the cells are included in the right-hand panels (bright field microscopy, 48× magnification).

Impairment of metabolism frequently affects cell growth. To test whether doxycycline concentrations regularly used in Tet-systems have an effect on cell density, we performed growth analyses under serum-containing (10% FCS) culture conditions without glucose restriction (25 mM glucose). Concentrations up to 1 µg/mL doxycycline had no effect on cell density (Figure 4). However, 10 µg/mL doxycycline reduced cell density in all tested cell lines (Figure 4). Morphologically, an increase in cellular detachment was not detectable (Figure 4, right panels). Additionally, under normoxic conditions we did not detect an increase in LDH release under doxycycline (Figure 3); therefore, a reduction in proliferation—as has been reported for other cell lines [14]—is a plausible explanation for the reduced cell density.

### 2.5. Doxycycline Concentrations of 0.1 and 1 µg/mL Are Sufficient for Full Induction of a Tet-System and Doxycycline-Mediated Effects on Metabolism in Tet-System-Transfected Glioma Cells Correspond to Those in Wild-Type Cells

**Figure 5:**
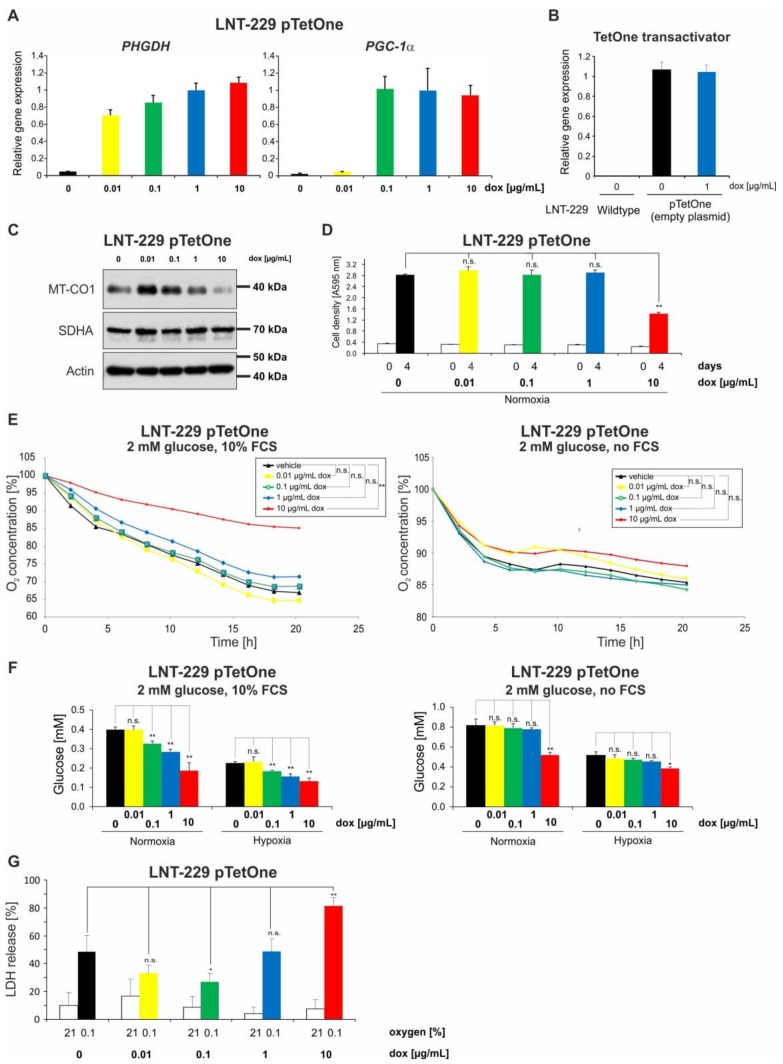
Dose-dependent effects of doxycycline on gene expression in pTetOne glioma cells and metabolic changes following treatment with doxycycline in Tet-system-transfected cells. (**A**) cDNA of LNT-229 pTetOne phosphoglycerate-dehydrogenase (PHGDH) and LNT-229 pTetOne peroxisome proliferator-activated receptor gamma coactivator 1 alpha (PGC-1α) cells cultured with vehicle, 0.01, 0.1, 1, or 10 µg/mL doxycycline for 24 h was generated. Gene expression of PGC-1α or PHGDH was quantified by qPCR; values are normalized to 18S as well as SDHA housekeeping gene expression (*n* = 3, mean ± S.D.). (**B**) cDNA of LNT-229 pTetOne cells without an inducible cDNA sequence cultured with either vehicle or 1 µg/mL doxycycline for 24 h and LNT-229 wild-type cells was generated. Gene expression of the TetOne transactivator gene was quantified by qPCR; values are normalized to 18S as well as SDHA housekeeping gene expression (*n* = 3, mean ± S.D.). (**C**) LNT-229 pTetOne cells without an inducible cDNA sequence were incubated in serum-containing (10% FCS) medium without glucose restriction (25 mM glucose) with vehicle, 0.01, 0.1, 1, or 10 µg/mL doxycycline under normoxic conditions (21% oxygen) for 24 h. Cellular lysates were analyzed by immunoblot with antibodies for MT-CO1, SDHA, and actin. (**D**) LNT-229 pTetOne cells without an inducible cDNA sequence were incubated in serum-containing (10% FCS) culture conditions without glucose restriction (25 mM glucose) with vehicle, 0.01, 0.1, 1, or 10 µg/mL doxycycline for 4 days under normoxia (21% oxygen). Cell density was measured by CV staining at the beginning of cultivation and after 4 days (*n* = 4, mean ± S.D.; n.s. = not significant, ** *p* < 0.01). (**E**) LNT-229 pTetOne cells without an inducible cDNA sequence were incubated in either glucose-restricted (2 mM glucose) DMEM containing 10% FCS (left panel) or glucose-restricted (2 mM glucose) serum-free DMEM (right panel) in each condition with vehicle, 0.01, 0.1, 1, or 10 µg/mL doxycycline and overlaid with sterile paraffin oil. Oxygen consumption was measured by a fluorescence-based assay and is depicted relative to the start of the experiment (*n* = 3, mean; n.s. = not significant, * *p* < 0.05, ** *p* < 0.01; for easier distinction of the curves, the standard deviation of values has been omitted in the diagrams). (**F**) LNT-229 pTetOne cells without an inducible cDNA sequence were incubated either in glucose-restricted (2 mM glucose) DMEM containing 10% FCS (left panel) or glucose-restricted (2 mM glucose) serum-free DMEM (right panel) in each condition with vehicle, 0.01, 0.1, 1, or 10 µg/mL doxycycline under normoxic conditions (21% oxygen) or 0.1% oxygen for 6 h. Glucose consumption was determined in the supernatant (*n* = 3, mean ± S.D.; n.s. = not significant, * *p* < 0.05, ** *p* < 0.01). (**G**) LNT-229 pTetOne cells without an inducible cDNA sequence were exposed to glucose-restricted (2 mM glucose) serum-free DMEM with vehicle, 0.01, 0.1, 1, or 10 µg/mL doxycycline under normoxic conditions or 0.1% oxygen until cell death was observed. Cell death was quantified using the LDH release assay (*n* = 4, mean ± S.D.; n.s. = not significant, * *p* < 0.05, ** *p* < 0.01).

To test doxycycline dose-dependent gene expression in a modern Tet-on system, we used the TetOne 3G system with two different inducible sequences. LNT-229 pTetOne cells were treated with doxycycline for 24 h. Expression of target genes was measured by qPCR. Doxycycline induced expression of *phosphoglycerate-dehydrogenase* (*PHGDH*) in LNT-229 pTetOne PHGDH cells in a dose-dependent manner starting with a concentration of 0.01 µg/mL (Figure 5A). In LNT-229 pTetOne PPARGC1A/PGC-1α (peroxisome proliferator-activated receptor gamma coactivator 1 alpha cells, gene expression was induced with 0.1 µg/mL doxycycline. A dose-dependent further increase in *PGC-1α* gene expression was not detectable (Figure 5A). To test the observed metabolic effects also in cells transfected with a Tet-system, we analyzed LNT-229 cells transfected with the pTetOne plasmid without a cDNA cloned after the Tet-responsive promoter (LNT-229 pTetOne). qPCR confirmed the expression of the TetOne transactivator gene, which was not affected by doxycycline treatment (Figure 5B). Immunoblot analyses confirmed that MT-CO1 protein content was also decreased with doxycycline concentrations of 1 µg/mL and above in LNT-229 pTetOne cells. A concentration of 10 µg/mL doxycycline rendered MT-CO1 almost undetectable in these cells (Figure 5C). Growth analyses under serum-containing (10% FCS) culture conditions without glucose restriction (25 mM glucose) confirmed that concentrations up to 1 µg/mL doxycycline had no effect on cell density whereas 10 µg/mL doxycycline reduced cell density (Figure 5D). Furthermore, a dose-dependent reduced oxygen consumption could be observed (Figure 5E). Under FCS-containing conditions, a significant effect on oxygen consumption was observed for 10 µg/mL (Figure 5E, left panel). Upon serum withdrawal, impaired oxygen consumption was detectable, but it was not statistically significant (Figure 5E, right panel). A potentially compensatory induction of glucose consumption was also observed in LNT-229 pTetOne cells in a doxycycline dose-dependent manner (Figure 5F). A significant increase in glucose consumption was observed with 0.1, 1, and 10 µg/mL doxycycline under incubation with FCS in normoxia and hypoxia (Figure 5F, left panel). Upon serum withdrawal, an increase in glucose consumption could be observed with 10 µg/mL doxycycline under normoxic and hypoxic conditions (Figure 5F, right panel). We furthermore performed cell viability measurements. A doxycycline concentration of 0.1 µ/mL protected LNT-229 pTetOne cells from hypoxia-induced cell death. (Figure 5G). In contrast, a further increased concentration of 10 µg/mL doxycycline enhanced sensitivity to hypoxia-induced cell death as indicated by an increase in LDH release (Figure 5G). Under normoxic conditions all doxycycline doses had no effect on LDH release (Figure 5G).

### 2.6. Doxycycline Impairs Mitochondrial Function and Alters Metabolism of Astroglial SVG Cells

**Figure 6:**
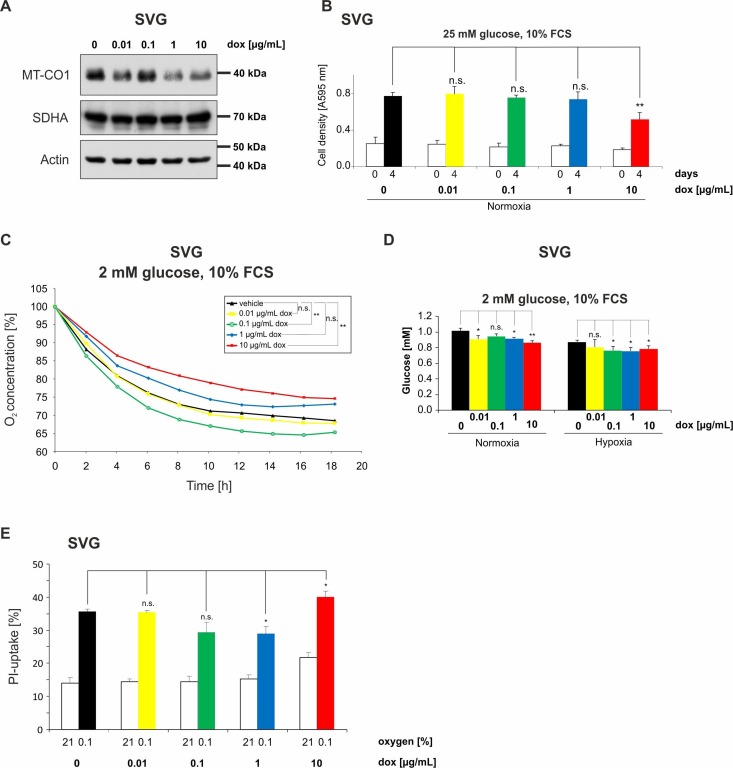
Effects of doxycycline on SVG cells. (**A**) SVG cells were incubated in serum-containing (10% FCS) medium without glucose restriction (25 mM glucose) with vehicle, 0.01, 0.1, 1, or 10 µg/mL doxycycline under normoxic (21% oxygen) conditions for 24 h. Cellular lysates were analyzed by immunoblot with antibodies for MT-CO1, SDHA, and actin. (**B**) SVG cells were incubated in serum-containing (10% FCS) culture conditions without glucose restriction (25 mM glucose) with vehicle, 0.01, 0.1, 1, or 10 µg/mL doxycycline for 4 days. Cell density was measured by CV staining at the beginning of cultivation and after 4 days (*n* = 4, mean ± S.D.; n.s. = not significant, ** *p* < 0.01). (**C**) SVG cells were incubated in glucose-restricted (2 mM glucose) DMEM containing 10% FCS with vehicle, 0.01, 0.1, 1, or 10 µg/mL doxycycline and overlaid with sterile paraffin oil. Oxygen consumption was measured by a fluorescence-based assay and the oxygen concentration is depicted relative to the start of the experiment (*n* = 3, mean; n.s. = not significant, ** *p* < 0.01; for easier distinction of the curves, the standard deviation of values has been omitted in the diagrams). (**D**) SVG cells were incubated in glucose-restricted (2 mM glucose) DMEM containing 10% FCS with vehicle, 0.01, 0.1, 1, or 10 µg/mL doxycycline under normoxic conditions or 0.1% oxygen for 6 h. Glucose consumption was determined in the supernatant (*n* = 3, mean ± S.D.; n.s. = not significant, * *p* < 0.05, ** *p* < 0.01). (**E**) SVG cells were exposed to glucose-restricted (2 mM glucose) serum-free DMEM with 0, 0.01, 0.1, 1, or 10 µg/mL doxycycline under normoxic conditions (21% oxygen) or 0.1% oxygen until cell death was observed. Cell death was quantified by propidium iodide staining (*n* = 3, mean ± S.D.; n.s. = not significant, * *p* < 0.05).

To also test the observed metabolic effects in nontumor cells, the immortalized astroglial cell line SVG was analyzed. MT-CO1 protein content was also decreased with doxycycline concentrations of 1 µg/mL and higher in these cells (Figure 6A). Growth analyses under serum-containing (10% FCS) culture conditions without glucose restriction (25 mM glucose) confirmed also in SVG cells that concentrations up to 1 µg/mL doxycycline had no effect on cell density whereas 10 µg/mL doxycycline reduced cell density (Figure 6B). Furthermore, a dose-dependent reduced oxygen consumption could be observed (Figure 6C). Under FCS-containing conditions, a significant effect on reduction of oxygen consumption was observed for 10 µg/mL (Figure 6C). An increase in glucose consumption was also observed in SVG cells in a doxycycline dose-dependent manner (Figure 6D). A significant increase in glucose consumption was observed with 0.01, 1, and 10 µg/mL doxycycline under incubation with FCS in normoxia and with 0.1, 1, and 10 µg/mL in hypoxia (Figure 6D). Similar to the effects observed in glioma cells, 1 µg/mL doxycycline protected from and 10 µg/mL doxycycline sensitized to hypoxia-induced cell death (Figure 6E), as indicated by the corresponding changes in propidium iodide staining.

## 3. Discussion

In our study, we investigated dose-dependent metabolic changes following doxycycline treatment in three wild-type glioma cell lines (LNT-229, G55 and U343), LNT-229 pTetOne cells transfected with the pTetOne vector, and the astroglial cell line SVG. This is of particular importance because doxycycline is the main agent used for inducible gene expression systems, and metabolism is becoming more and more recognized as a target in cancer therapy. Our investigations confirmed the ability of doxycycline to cause severe mitonuclear protein imbalance in glioma and astroglial cells, especially at high concentrations (Figure 1A, Figure S1, Figure 5C and Figure 6A). Lower concentrations of doxycycline were sufficient to impair the oxygen consumption rate of all tested cell lines (Figure 1B–D, Figure 5E and Figure 6C). We further observed an increase in glucose consumption as a potential compensatory mechanism (Figure 2, Figure 5F and Figure 6D). Doxycycline had no effect on cell growth at concentrations regularly used to induce gene expression. Only the highest tested concentration of doxycycline (10 µg/mL) impaired cell growth (Figure 4, Figure 5D and Figure 6B). Furthermore, doxycycline led to a dose-dependent protection from hypoxia-induced cell death up to 1 µg/mL, with a further increase then displaying an opposite sensitization of glioma cells to hypoxia-induced cell death (Figure 3, Figure 5G and Figure 6E). It is interesting to speculate about the underlying mechanisms for the protection from hypoxia-induced cell death mediated by 0.1 and 1 µg/mL doxycycline depending on the cell line. Under such conditions, cells frequently displayed a reduction of oxygen consumption whereas the mitonuclear protein imbalance was not as pronounced as under 10 µg/mL doxycycline. The reduction in oxygen consumption could be one mechanism which triggers adaptive responses to cope with a reduced oxygen supply. Further reduced oxygen consumption could correlate with reduced production or release of potentially toxic reactive oxygen species. Increases in reactive oxygen species are a known phenomenon under hypoxia [17]. The converse sensitization of cells to hypoxia-induced cell death by 10 µg/mL doxycycline might be due to a potentially higher degree of mitochondrial dysfunctionality, e.g., with increased production of reactive oxygen species, mediated by the more pronounced mitonuclear protein imbalance (Figure 1A and Figure 5C). In summary, doxycycline may cause confounding effects of relevant strength. Caution in interpreting results obtained with Tet-systems, especially when studying metabolism, is therefore mandated. For the initially reported Tet-systems, concentrations of 0.1–0.5 µg/mL doxycycline were commonly used [18]. For the newer generation of systems, concentrations of 0.01–0.2 µg/mL are usually sufficient for target gene induction or repression [19]. Our results emphasize the necessity to control for doxycycline-dependent metabolic effects and to titrate the lowest effective doxycycline concentration which may be dependent on cell type, clone, and transcriptional target. In our experiments, we observed effects with concentrations as little as 0.1 µg/mL doxycycline; therefore, this concentration appears a plausible upper limit to minimize metabolic effects with regard to hypoxia-induced cell death as well as glucose consumption.

The effect of doxycycline on mitochondria is most likely fundamental and regardless of the cell type the mitochondria reside in. While other cell types are likely affected in a similar manner [14,20], the relevance for proliferating cancer cells could be more distinct because cancer cells frequently rely on mitochondrial metabolites beyond ATP, e.g., mitochondrial folate synthesis, to sustain tumor growth. Further, the conditions employed in our experiments with glucose and oxygen restriction mirror conditions that can be found in the tumor microenvironment. Therefore, an increased sensitivity of cancer cells (especially in an in vivo setting) for doxycycline-mediated effects appears plausible and as part of a metabolic treatment approach, doxycycline could be an interesting component. Different studies on the therapeutic potential of doxycycline in human tumor xenografts and other animal models have shown anti-tumor effects [21,22]. Recent articles also reported a potential treatment approach for the selective targeting of cancer stem cells [23,20]. Clonal expansion and survival of cancer stem cells across different tumor entities can rely on a conserved dependence on mitochondrial biogenesis. In this context, different classes of antibiotics including tetracyclines with known “side effects” on mitochondrial biogenesis were able to eradicate cancer stem cells in a panel of different cancer cell lines and tumor types including glioblastoma [20]. Advantages of this treatment strategy are, on the one hand, that targeting mitochondrial biogenesis seems to be a “mutation-independent” approach and, on the other hand, that doxycycline appears to have no relevant toxicity under the tested concentrations and, further, has a well-known safety profile in humans [20].

## 4. Materials and Methods

### 4.1. Reagents, Cell Lines, and Culture Conditions

Doxycycline and reagents not specified were purchased from Sigma (Taufkirchen, Germany). LNT-229 cells were a kind gift from Nicolas de Tribolet (Lausanne, Switzerland) [24,25]; G55 cells were a kind gift from Manfred Westphal and Kathrin Lamszus (Hamburg, Germany) [26]. U343 cells were kindly provided by Donat Kögel (Frankfurt am Main, Germany). SVG cells were purchased from the American Type Culture Collection (ATCC, Manassas, VA, USA). Cell lines were maintained at 37 °C in a cell culture incubator (Binder, Tuttlingen, Germany) under a CO_2_ atmosphere (5%) in DMEM containing 10% FCS (Biochrom KG, Berlin, Germany), 100 IU/mL penicillin, and 100 µg/mL streptomycin (Life Technologies, Karlsruhe, Germany). Doxycycline was dissolved in water (stock 1 mg/mL). The following concentrations were analyzed: 0.0, 0.1, 1, 10 µg/mL. Cell growth using crystal violet staining (CV) was measured after incubation under standard conditions. All other assays were performed either in DMEM containing 2 mM glucose without FCS or in DMEM containing 2 mM glucose and 10% FCS as specified. When comparing doxycycline-treated cells, equal cell densities were confirmed by CV in a parallel assay [24,27].

### 4.2. Generation of pTetOne Cells

The pTetOne system was purchased from Takara Clontech (#634301; Saint-Germain-en-Laye, France). Cloning of the PHGDH and PGC-1α sequences was done by Genscript (Piscataway Township, NJ, USA) using the NotI and MluI restriction sites. Cell transfections were performed using the Xfect Transfection Reagent (Takara Clontech, Saint-Germain-en-Laye, France). One day before transfection, 130,000 cells were seeded in 6-well dishes in order to achieve 50–70% confluence the day after. The transfection was conducted following the manufacturer’s protocol. For selection, the Linear Puromycin Marker (Takara Clontech, Saint-Germain-en-Laye, France) was used.

### 4.3. Induction of Hypoxia

Hypoxia was induced as described previously [24,17]. Cells were seeded and allowed to attach in DMEM containing 10% FCS overnight under normoxia. Subsequently, the medium was removed and the cells were incubated in serum-free DMEM containing 2 mM glucose under normoxia or 0.1% oxygen. Conditions of 0.1% oxygen were induced by incubation in Gas Pak pouches as described elsewhere (Becton-Dickinson, Heidelberg, Germany) [28].

### 4.4. RNA Extraction and Quantitative Reverse Transcription-PCR (qPCR) Analysis

The qPCR protocol has previously been described [29]. For RNA purification, TRIzol^®^ and the EXTRACTME Kit (Blirt, Gdansk, Poland) were used. CDNA was synthesized using the Vilo cDNA synthesis kit (Invitrogen, Carlsbad, CA, USA) (10 min at 25 °C followed by 2 h at 42 °C). The reaction was stopped at 85 °C for 10 min. qPCR was performed with the IQ5 real-time PCR detection system (Biorad, Munich, Germany) using ABsolute Blue SYBR Green Fluorescein Q-PCR Mastermix (Thermo Fisher Scientific, Hamburg, Germany). The employed primer pairs are summarized in Table 1. 18S and SDHA were used as housekeeping genes for normalization. Cycle threshold (*C*t) values were normalized for amplification of the 18S. The data were analyzed using the Vandesompele method [30].

### 4.5. Lysate Preparation and Immunoblot Analysis

After incubation, cells were washed with ice-cold PBS and immediately frozen in liquid nitrogen. Lysates were prepared using RIPA Lysis and Extraction Buffer (Thermo Fisher Scientific, Hamburg, Germany) consisting of 25 mM Tris-HCl pH 7.6, 150 mM NaCl, 1% NP-40, 1% sodium deoxycholate, and 0.1% SDS, with the addition of 1% Halt™ Protease and Phosphatase Inhibitor Single-Use Cocktail (Thermo Fisher Scientific, Hamburg, Germany), diluted in Laemmli buffer, subjected to SDS-PAGE analysis, and blotted to nitrocellulose membranes (0.45 µm; GE Healthcare, Little Chalfont, UK) by wet blotting [24]. Equal loading was ascertained by Ponceau staining. The membranes were blocked with Roti-Block (Roth, Karlsruhe, Germany) for 1 h. Afterwards, membranes were incubated with antibodies to MT-CO1 (#ab14705, 1:2000; Abcam, Cambridge, UK), SDHA (#11998, 1:1000; Cell Signaling Technology, Danvers, MA, USA) and actin (#sc-1616, 1:2000; Santa Cruz Biotechnology, Santa Cruz, CA, USA) in Roti-Block diluted 1:10 in water over night. The actin antibody detects α, β, and γ isoforms of actin. The secondary anti-mouse and anti-goat antibodies were purchased from Santa Cruz Biotechnology (#sc-516102 and #sc-2020). The secondary anti-rabbit antibody was purchased from Jackson ImmunoResearch (#111-036-144; West Grove, PA, USA). Membranes were in incubated in secondary antibodies diluted (anti-mouse 1:3000, anti-rabbit 1:10,000, and anti-goat 1:5000) in 1:10 Roti-Block for 1 h. For detection, chemiluminescence solution composed of 1 mL solution A (200 mL 0.1 M Tris-HCl pH 8.6, 50 mg luminol), 100 µL solution B (11 mg p-hydroxy coumaric acid, 10 mL DMSO), and 0.3 µL H_2_O_2_ (30%) was used. Quantification of protein bands was performed by measuring the pixel density with ImageJ software (National Institutes of Health; Bethesda, MD, USA) on scanned films.

### 4.6. Cell Density and Cell Viability Assays

Cell density was measured by crystal violet (CV) staining as described previously [27,31]. Cell viability analysis by lactate dehydrogenase (LDH) release assay was performed with the Cytotoxicity Detection Kit (LDH) (Roche, Mannheim, Germany) as described previously [17]. Briefly, for quantification of cell death, LDH activity was measured first in the cell supernatant and again after cell lysis to calculate the ratio (LDH release) as a marker of cell death. Because the activity of LDH is put into relation within the same sample, a potential regulation of LDH expression by experimental conditions should not have an effect of the ratio. Cell viability measurement by propidium iodide (PI) uptake and flow cytometry has recently been described [24,17]. Analysis was performed by flow cytometry employing a BD Canto II flow cytometer and BD FACS Diva software version 6.1.3 (BD Biosciences, Franklin Lakes, NJ, USA).

### 4.7. Measurement of Glucose

The protocol for the measurement of glucose in the supernatant has also been described [17]. In brief, cell-free supernatant was collected. Afterwards glucose concentration was measured using the biochemistry analyzer Hitachi 917 (Roche, Basel, Swizerland).

### 4.8. Oxygen Consumption

Measurement was performed as previously described [17]. Cultured cells were carefully overlaid with sterile paraffin oil and oxygen consumption was measured with a fluorescence-based assay (PreSens, Regensburg, Germany). Briefly, this assay employs special 24-well dishes with an oxygen sensor at the bottom of each well (OxoDish, PreSens, Regensburg, Germany). A sensor dish reader noninvasively reads out and records the oxygen concentration at prespecified time intervals.

### 4.9. Statistical Analysis

Quantitative data are expressed as indicated including standard deviation (S.D.). The *p*-values were derived from two-tailed Student’s *t*-tests (Excel version 2010, Microsoft, Seattle, WA, USA) for all calculations except for oxygen consumption analysis, for which a nonlinear regression analysis followed by ANOVA was used (GraphPad Prism 7, GraphPad Software, LaJolla, CA, USA). For our purposes, *p*-values of *p* < 0.01 and *p* < 0.05 were considered highly significant (**) and significant (*), repectively, and values of *p* > 0.05 were considered not significant (n.s.).

## Figures and Tables

**Table 1:** Primer pairs for qPCR analysis.

Gene	Fwd	Rev
18S	5′-CGGCTACCACATCCAAGGAA-3′	5′-GCTGGAATTACCGCGGCT-3′
SDHA	5′-TGGGAACAAGAGGGCATCTG-3′	5′-CCACCACTGCATCAAATTCATG-3′
PGC-1α	5′-TCTGAGTCTGTATGGAGTGACAT-3′	5′-CCAAGTCGTTCACATCTAGTTCA-3′
PHGDH	5′-CTGCGGAAAGTGCTCATCAGT-3′	5′-TGGCAGAGCGAACAATAAGGC-3′
TetOne transactivator	5′-CTATGCCCCCACTTCTGAAA-3′	5′-GTCAGCAGGCAGCATATCAA-3′

## Supplementary Materials

The following are available online at http://www.mdpi.com/1422-0067/19/5/1504/s1.

## Abbreviations

CV	Crystal violet
FCS	Fetal calf serum
LDH	Lactate dehydrogenase
MT-CO1	Mitochondrially-encoded cytochrome c oxidase I
PHGDH	Phosphoglycerate-dehydrogenase
PPARGC1A/PGC-1α	Peroxisome proliferator-activated receptor gamma coactivator 1-alpha
SDHA	Succinate dehydrogenase complex, subunit A
Tet-inducible systems	Tetracycline-inducible systems
