# Supplementary material for: Doxycycline Impairs Mitochondrial Function and Protects Human Glioma Cells from Hypoxia-Induced Cell Death: Implications of Using Tet-Inducible Systems
Source: Int J Mol Sci. 2018 May 17;19(5):1504. doi: 10.3390/ijms19051504 (PMC5983704; doi:10.3390/ijms19051504)
Supplement: Supplementary file 1 [file ijms-19-01504-s001.pdf]

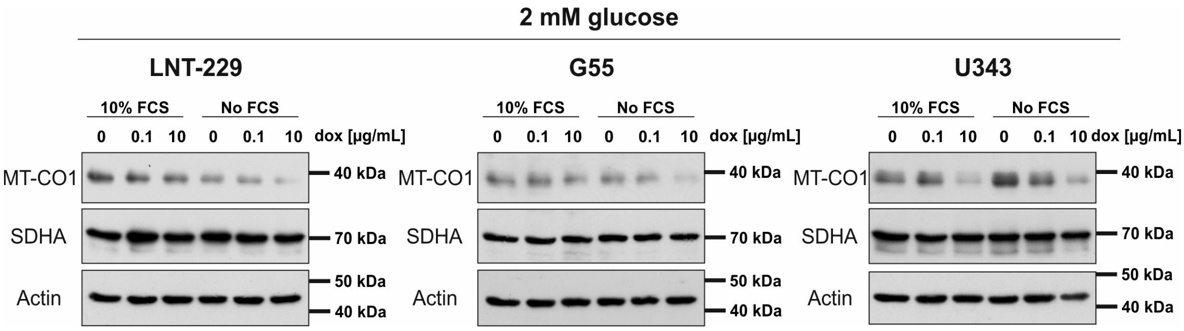

Figure S1: Doxycycline causes mitonuclear protein imbalance in glioma cells under low glucose conditions. LNT-229, G55 and U343 glioma cells were incubated in serum containing (10% FCS) or serum-free (No FCS) medium with glucose restriction (2 mM glucose) with vehicle, 0.1 or 10 µg/ml doxycycline under normoxic conditions for 24 h as indicated. Cellular lysates were analyzed by immunoblot with antibodies for MT-CO1, SDHA and actin.
